# Post-marketing safety of lecanemab: a real-world study based on FAERS database, multicenter cohort and network pharmacology

**DOI:** 10.3389/fpsyt.2026.1822543

**Published:** 2026-06-22

**Authors:** Xiaoxuan Xing, Ke Wang, Yingnan Feng, Chao Wu, Lihua Jia, Huiying Li, Zhiyong Wen, Yinan Tang, Zhizhou Wang, Xiaotong Zhang, Xiaoxi Li, Yiming Hua, Lan Zhang, Xianzhe Dong

**Affiliations:** 1 Department of Pharmacy, Xuanwu Hospital of Capital Medical University, Beijing, China; 2 Department of Pharmacy, Peking University International Hospital, Beijing, China; 3 Department of Neurology, Beijing United Family Hospital, Beijing, China; 4 Department of Pharmacy, Zhuhai People’s Hospital (The Affiliated Hospital of Beijing Institute of Technology, Zhuhai Clinical Medical College of Jinan University), Zhuhai, China; 5 Department of Pharmacy, China-Japan Friendship Hospital, Beijing, China

**Keywords:** adverse events, Alzheimer’s disease, ARIA, FAERS, lecanemab, network pharmacology, pharmacovigilance, real-world study

## Abstract

**Introduction:**

Lecanemab is a monoclonal antibody targeting amyloid-beta (Aβ) approved for treating Alzheimer’s disease (AD) with mild cognitive impairment or mild dementia. Continuous monitoring of its real-world safety profile remains essential. This study aimed to analyze lecanemab-related adverse events (AEs) using the FDA Adverse Event Reporting System (FAERS) database and a multicenter cohort, and explored mechanisms via network pharmacology.

**Methods:**

We conducted an updated disproportionality analysis of FAERS data from 2023 to 2024 to identify disproportionate reporting signals (SDRs). A multicenter retrospective cohort study was performed, including lecanemab users from June 2024 to February 2025. Data were collected from electronic medical records of five tertiary hospitals. AEs were identified and influencing factors of AE occurrence were analyzed. Additionally, a drug-gene interaction network was constructed to explore potential mechanisms.

**Results:**

In the FAERS analysis of 2,764 AEs from 1,389 lecanemab users, 12 of 41 positive SDRs were prioritized, with 75% (9/12) being nervous system disorders, primarily amyloid-related imaging abnormalities (ARIA). In the cohort study, 29.05% (43/148) of patients experienced AEs, with infusion-related reactions being most common. Age was identified as a risk factor for AE occurrence [OR (95% CI): 1.109 (1.011–1.215), P = 0.028], while pre-treatment significantly reduced AE incidence. Gene enrichment analysis suggested potential links between lecanemab-related genes and AEs.

**Discussion:**

This study provides real-world evidence on the risk profile of lecanemab, highlighting the importance of continued safety monitoring. These findings may inform discussions about the risks associated with anti-amyloid treatments and warrant further confirmation in large-scale prospective studies.

## 1 Introduction

Alzheimer’s disease (AD), the most prevalent dementia, causes progressive cognitive decline (1). Worldwide, 87 million people have early AD and 32 million have AD dementia (2). And by 2050, global AD cases are projected to exceed 115 million (3). In China, the prevalence of AD among individuals over the age of 65 is estimated at 6.6%, with rates doubling every five years and surpassing 22% in those aged 80 and above (4). The number of AD patients in China accounted for approximately 24% of the global total in 2019, bearing a substantial healthcare burden (5).

The two major pathological hallmarks of AD are the accumulation of senile plaques formed from amyloid-β (Aβ) peptides and the formation of neurofibrillary tangles composed of hyperphosphorylated tau proteins within the brains of AD patients (6). Traditional treatments include cholinesterase inhibitors (ChEIs) and N-methyl-D-aspartate (NMDA) receptor antagonists only alleviate symptoms (7). After numerous unsuccessful endeavors to create a disease‐modifying therapy for AD, three monoclonal antibody drugs targeting Aβ - aducanumab, lecanemab, and donanemab – have been successively approved for use (8, 9).

Lecanemab, a humanized immunoglobulin gamma 1 (IgG1) monoclonal antibody targeting aggregated Aβ, received US approval for mild cognitive impairment or mild dementia AD in January 2023 (10). It was approved in China (January 2024; clinical use from June 2024) and by the European Medicines Agency (EMA) (April 2025, following prior rejection). In clinical trials, the most common adverse events (AEs) of lecanemab were infusion-related reactions, Amyloid related imaging abnormalities (ARIA) with cerebral microhaemorrhages, cerebral macrohemorrhages, or superficial siderosis (ARIA-H), and ARIA with edema or effusions (ARIA-E) (11). However, it should be noted that clinical trials have inherent limitations, such as stringent eligibility criteria that exclude patients with comorbidities or those at elevated risk of AEs, potentially leading to underestimation of AE incidence. Furthermore, they often lack sufficient statistical power to detect rare events. In addition, FDA have issued a “black box” warning, highlighting the significant safety risks associated with ARIA (12). Given these concerns, it is essential to closely monitor the safety profile of lecanemab, particularly in real-world settings.

The FDA Adverse Event Reporting System (FAERS), a public voluntary AE database, was used in our prior study to identify potential new lecanemab-associated AEs through disproportionality analysis (detecting signals of disproportionate reporting) (13). Subsequently, several researchers have conducted observational studies on lecanemab AEs based on this database (14–18), and some have performed real-world cohort studies to investigate lecanemab-associated AEs (19, 20). However, to date, no study has integrated the above-mentioned database-derived disproportionality analyses with safety observations from real-world clinical practice. Moreover, the occurrence of AE varies among different ethnic groups. To further elucidate the real-world safety profile of lecanemab, we aim to update the disproportionality analysis using FAERS data and conduct a safety analysis on a multicenter cohort of patients receiving lecanemab treatment in this study. Additionally, the mechanisms underlying the AEs caused by lecanemab, especially ARIA, remain poorly understood. In this study, we will explore these mechanisms using network pharmacology from a bioinformatics perspective.

## 2 Methods

### 2.1 Identification of SDR in FAERS

We searched the reports submitted to FAERS between January 1, 2023, and December 31, 2024, with lecanemab designated as the “primary suspect drug”. Full details on data sourcing and vetting are in the **Supplementary Material**. We employed a case/non-case approach akin to a case-control study design (21). Cases were defined as reports involving lecanemab, while non-cases included AE reports for all other medications in FAERS (22, 23). Within this cohort, we assessed disproportionality: if the proportion of AEs of interest is higher in patients exposed to lecanemab (cases) compared to those not exposed (non-cases), an association between the medication and the event can be hypothesized, indicating a disproportionality signal (24).

We used two disproportionality approaches to increase consistency and robustness of findings. A SDR for an AE was identified when both disproportionality measures met the criteria. The reporting odds ratio (ROR) was considered statistically significant if the lower limit of the ninety-five percent confidence intervals (CIs) was greater than 1, while the Bayesian information component (IC) was deemed significant if the lower limit of the CI was above 0 (25). Further details on analysis are reported in the **Supplementary Material**. A semiquantitative scoring system incorporating five key parameters was implemented to prioritize SDR: number of target events, the lower limit of the 95% confidence interval of the ROR (ROR_025_), mortality proportion, important medical events (IMEs) or designated medical events (DMEs), relevant evidence evaluation (**Supplementary Table 1**) (26). SDRs achieving moderate or higher priority were designated as clinically consequential, necessitating enhanced pharmacovigilance monitoring. Time-to-onset (TTO) intervals were calculated as the duration between lecanemab initiation and adverse event manifestation. Medians, quartiles, and the Weibull shape parameter (WSP) test were used to assess the TTO. The cumulative distribution of TTO was plotted to describe the cumulative proportion of reported lecanemab-related AEs over time in different groups. We compared age, sex, number of medications (all medications the patients were taking) between reports with serious and non-serious outcomes. A “volcano map” was created to visualise differences in AE severity.

The analysis by therapeutic area was used to mitigate the effects of confounding by indication on SDR detection by limiting the analysis to a population of patients that could share common risk factors and diseases (27). Donanemab and aducanumab are aβ-amyloid-targeting monoclonal antibody mechanistically similar to lecanemab. We recalculated RORs and IC values for donanemab and aducanumab as a comparator, enabling cross-validation of lecanemab’s safety profiles.

### 2.2 Multicenter cohort analysis on AEs

We conducted a multicenter retrospective cohort study in five tertiary hospitals (Xuanwu Hospital of Capital Medical University, China-Japan Friendship Hospital, Beijing United Family Hospital, Peking University International Hospital, and Zhuhai People’s Hospital), all of which are general hospitals with specialized cognitive disorder clinics. Patients with AD either presented voluntarily to these clinics or were referred from other healthcare institutions at different levels. We included patients who were admitted to the hospital and used lecanemab from June 2024 to February 2025. The study inclusion criteria were 1) patients with the clinical diagnose of mild cognitive impairment (MCI) due to AD, or mild AD dementia; 2) patients with amyloid pathology confirmed by amyloid positron emission tomography (PET). The study excluded patients who had used lecanemab prior to their first admission to the study hospital for lecanemab treatment, to ensure that the safety profile after the first dose could be observed.

We obtained the study data from the hospitals’ electronic medical record systems. The following data were collected: the patient’s personal information (date of birth, gender, height, and weight, history of smoking or drinking, family history of cognitive impairment, and history of allergies), disease information (cognitive assessment scores including Mini-Mental State Examination (MMSE) score and Montreal Cognitive Assessment (MoCA) score, discharge diagnoses, and laboratory test results) and medication information (drug name, indications, dosage). We extracted the dosage, the infusion time of lecanemab used, as well as the names or symptoms, the time of occurrence, the management measures taken, and the time of resolution of AEs related to lecanemab from the medical records. We further confirmed the AEs based on the patients’ medication use. The AEs were coded according to the Medical Dictionary for Regulatory Activities (MedDRA 27.0). Two researchers independently assessed the causal relationship and severity of adverse events (AEs) related to lecanemab according the standard of China National Medical Products Administration (28). In cases of disagreement, a consensus was reached through discussion. Only AEs with a causal relationship rating of “possible”, “probable”, or “definite” were ultimately included. The aged-adjusted Charlson comorbidity index (aCCI) score was calculated based on the discharge diagnoses (29). Variables included age, gender, apolipoprotein E (ApoE) ϵ4 Status, dosage of lecanemab, number of diagnoses at discharge, aCCI score, number of drugs used simultaneously and preventative medications (diphenhydramine) using were used to analyze the influencing factors of AEs occurrence. In addition, the use of concomitant psychotropic medications (antidepressants, antipsychotics, and benzodiazepines) was recorded and analyzed for association with AEs.

Descriptive statistics were used to describe the general characteristics of the study population and occurrence of AEs. For normally distributed continuous data, the results were expressed as mean ± standard deviation, and comparisons between groups were made using T-tests. For data that did not follow a normal distribution, the results were described using the median (M) and 25 and 75 percentiles (P25, P75), and comparisons were made using the Wilcoxon signed-rank test. Categorical data were presented as counts and proportions (%), and comparisons between groups were conducted using the chi-square test (χ² test) or Fisher’s exact test. A logistic regression model was used to analyze the influencing factors of AEs occurrence. Odds ratios (OR) and 95% confidence intervals (95% CI) were derived from this model. A two-sided P value<0.05 was considered statistically significant. All statistical analyses were performed using SAS software (version 9.4).

### 2.3 Network pharmacological analysis

We utilized biomedical databases, including four drug-target interaction databases, BioGRID (https://thebiogrid.org/), TTD (https://db.idrblab.net/ttd/), DrugBank (https://go.drugbank.com/) and STRING (https://cn.string-db.org/), to predict targets potentially interacting with amyloid precursor protein (APP) and establish a candidate target set for lecanemab (L set). In parallel, disease-associated databases, including five disease-associated databases, CTD (https://ctdbase.org/), GeneCards (https://www.genecards.org/), MalaCards (https://www.malacards.org/), OMIM (https://omim.org/), Open Targets Platform (https://www.targetvalidation.org) were employed to retrieve and collate targets associated with ARIA-E or ARIA-H, constituting an adverse event target set (AE set). The intersection between the L set and AE set was defined as the lecanemab-ARIA-E/H potential target set (L-A set). The L-A set was subjected to Drug-Target gene Interaction (DTI) network analysis via Cytoscape software, followed by functional enrichment analyses using two R packages (DOSE and clusterProfiler) covering Disease Ontology (DO), Gene Ontology (GO), and Kyoto Encyclopedia of Genes and Genomes (KEGG) pathways to elucidate its biomedical significance.

## 3 Results

### 3.1 Disproportionality analyses

Throughout the study period, a total of 2,764 AE reports by 1,389 patients were obtained from the FAERS database following the exclusion of duplicates. The clinical characteristics of these patients are outlined in **Table 1**. Of the 41 AE terms with positive SDR, all of which had at least 4 reported cases, the most frequently reported AEs included headache (n=234, 13.34%), ARIA-E (n=190, 10.83%), ARIA-H (n=164, 9.35%), chills (n=138, 7.87%), infusion-related reaction (n=120, 6.84%), fatigue (n = 105, 5.99%), pyrexia (n = 82, 4.68%) and confusional state (n = 80, 4.56%) (**Supplementary Table 2**). The results of the disproportionality analysis for AEs among lecanemab users are presented in **Supplementary Table 3**. AEs exhibited ROR_025_ values ranging from 1.54 (fall) to 25290.04 (ARIA). According to the clinical priority assessment, 12 (29%) AEs were identified as having moderate clinical priority (**Table 2**), categorized into 3 groups: nervous system disorders, general disorders and administration site conditions, injury, poisoning and procedural complications. The ARIA-E (n=190, ROR_025_ = 16701.18) and ARIA-H (n=164, ROR_025_ = 16987.36) were assigned a top priority score of 7. **Supplementary Table 4** shows the 2×2 contingency tables for ARIA−E and ARIA−H.

**Table 1:** Characteristics of the patients submitted to the US FDA adverse event reporting system for lecanemab.

Characteristics	All adverse events No (%)	ARIA no (%)
Gender		
Female	720(51.84)	176(56.05)
Male	524(37.72)	89(28.34)
Not Specified	145(10.44)	49(15.61)
Age, y		
18-44	0(0.00)	0(0.00)
45-64	3(0.22)	25(7.96)
65-74	123(8.86)	96(30.57)
≥75	896(64.51)	104(33.12)
Not Specified	367(26.42)	89(28.34)
Reporting year		
2023	193(13.89)	28(8.92)
2024	1196(86.11)	286(91.08)
Reporter		
Consumer	523(37.65)	51(16.24)
Physician	485(34.92)	174(55.41)
Pharmacist	349(25.13)	80(25.48)
Not Specified	32(2.30)	9(2.87)
Reporting country		
United States of America	1237(89.06)	279(88.85)
Japan	111(7.99)	20(6.37)
China	16(1.15)	4(1.27)
Others	25(1.80)	11(3.50)
Patients with serious and nonserious reports		
Non-Serious	955(68.75)	188(59.87)
Serious	434(31.25)	126(40.13)
Reported outcome^a^		
Death	55(3.96)	11(3.50)
Nonfatal^b^	302 (21.74)	98(31.21)
Nonfatal^c^: other serious outcome	145 (10.44)	47(14.97)
Time-to-onset, d^c^		
N (Missing)	804(585)	169(145)
Mean (SD)	64.36(178.87)	111.57(207.28)
Median (Q1, Q3)	1.00(0.00,48.00)	49.00 (38.00, 78.00)
Min,Max	0.00,1283.00	0.00,1260.00

ARIA, Amyloid related imaging abnormality.

^a^ The patients can have multiple serious AE outcomes.

^b^ Hospitalization, disability, life-threatening, or required intervention.

^c^ The time from the start of therapy to the date the event occurred.

**Table 2:** Clinical priority assessing results of SDR.

SOCs	PTs	n	ROR_025_	Death	IME/DME	Relevant evidence evaluation	Priority level (score)
Nervous system disorders	ARIA-E	190	16701.18	9	IMEs	++	moderate (7)
	ARIA-H	164	16987.36	8	IMEs	++	moderate (7)
	Headache	234	6.75	0	NA	++	moderate (6)
	Cerebral haemorrhage	28	9.29	6	IMEs	++	moderate (6)
	Brain oedema	15	12.42	1	IMEs	++	moderate (6)
	Status epilepticus	5	4.95	3	IMEs	++	moderate (6)
	ARIA	48	15844.35	0	NA	++	moderate (5)
	Seizure	22	2.14	3	IMEs	++	moderate (5)
	Cerebral microhaemorrhage	9	273.3	0	IMEs	++	moderate (5)
General disorders and administration site conditions	Chills	138	17.93	1	NA	++	moderate (6)
	Pyrexia	82	3.55	1	NA	++	moderate (5)
Injury, poisoning and procedural complications	Infusion related reaction	120	29.6	2	NA	++	moderate (6)

SOC, system organ class; SDR, Signals of disproportionate reporting; PTs, preferred terms; DMEs, designated medical events; lMEs, important medical events; ROR_025_, the lower limit of the 95% confidence interval of the Reporting Odds Ratio; ARIA, Amyloid related imaging abnormalities; ARIA-E, amyloid-related imaging abnormality with oedema/effusion; ARIA-H, amyloid-related imaging abnormality with cerebral microhaemorrhage, cerebral macrohaemorrhages, or superficial siderosis. n, number of cases, NA: not designated medical events or important medical events.

### 3.2 Time-to-onset analysis

**Figure 1:**
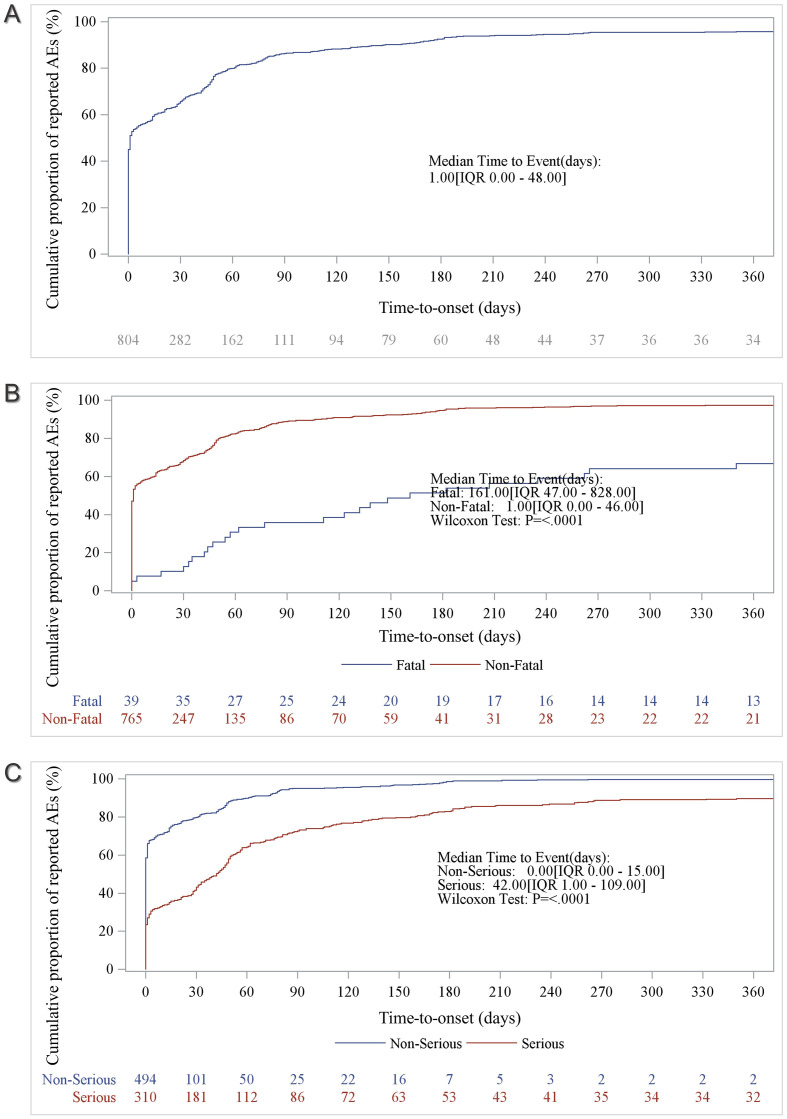
Time to onset of adverse events **(A)** overall, **(B)** difference between fatal and non-fatal and **(C)** difference between serious and non-serious of lecanemab. The time-to-onset difference between fatal and non-fatal or serious and non-serious adverse events was analyzed using the Wilcoxon Test.

Over 80% of AEs occurred within the first three months of treatment, with a median onset time of 1 days (0, 48). The median onset time was significantly longer in the fatal group than in the non-fatal group (Days: 161 vs. 1, P <0.0001). A similar trend was observed when comparing serious AEs and non-serious AEs (Days: 42 vs. 0, P<0.0001) (**Figure 1**). However, age, gender had no significant impact on the onset time of AEs (**Supplementary Figure 1**).

The results of the TTO and WSP analyses for SDR with moderate clinical priorities are summarized in **Table 3**. Most events, such as cerebral hemorrhage (β=0.94, 0.66–1.35) and seizures (β=1.26, 0.85–1.86), demonstrated random failure (β ≈1 or 95% CI overlapping 1). Early failure (β <1 with 95% CI upper limits <1) was observed in ARIA-E (β=0.80, 95% CI:0.71–0.91), headache (β=0.60, 0.48–0.74), chills (β=0.52, 0.35–0.76), and infusion-related reactions (β=0.52, 0.38–0.71). Wearout failure (β >1 with 95% CI lower limits >1) occurred exclusively in status epilepticus (β=7.86, 95% CI:2.47–24.94).

**Table 3:** Time-to-onset analysis for important SDR.

Categories	Cases, n	TTO (d) [median(Q1,Q3)]	Weibull distribution				Failure type
			β	95% CI	α	95% CI	
ARIA-E	190	49.00(40.00,76.00)	0.80	0.71-0.91	110.76	86.49-141.85	Early failure
ARIA-H	164	48.00(35.00,79.00)	0.92	0.80-1.07	93.04	73.10-118.43	random failure
Headache	234	0.00(0.00,1.00)	0.60	0.48-0.74	19.24	11.74-31.54	Early failure
Cerebral haemorrhage	28	54.00(27.00,177.00)	0.94	0.66-1.35	124.01	72.82-211.20	random failure
Brain oedema	15	45.50(30.00,62.00)	1.44	0.70-2.93	48.28	27.15-85.83	random failure
Status epilepticus	5	49.50(42.00,57.00)	7.86	2.47-24.94	52.77	43.80-63.57	wearout failure
ARIA	48	42.50(32.00,55.00)	0.80	0.51-1.24	86.83	36.19-208.35	random failure
Seizure	22	35.00(29.00,57.00)	1.26	0.85-1.86	49.15	32.19-75.03	random failure
Cerebral microhaemorrhage	9	25.00(0.00,211.00)	1.12	0.35-3.57	123.09	33.52-452.04	random failure
Chills	138	0.00(0.00,0.00)	0.52	0.35-0.76	13.31	4.35-40.67	Early failure
Pyrexia	82	0.00(0.00,0.00)	0.76	0.42-1.37	9.93	3.53-27.91	random failure
Infusion related reaction	120	0.00(0.00,1.00)	0.52	0.38-0.71	17.82	7.24-43.85	Early failure

SDR, Signals of disproportionate reporting; CI, confidence interval; ARIA, Amyloid related imaging abnormalities; ARIA-E, amyloid-related imaging abnormality with oedema/effusion; ARIA-H, amyloid-related imaging abnormality with cerebral microhaemorrhage, cerebral macrohaemorrhages, or superficial siderosis.

### 3.3 Serious versus non-serious AEs

The univariate analysis revealed that age (P = 0.020) and the number of medications (P<0.0001) were significantly associated with the serious AEs, whereas sex showed no significant association (P = 0.316). In the multivariate model adjusted for all three variables, age (P = 0.026) and the number of medications (P<0.0001) remained independent predictors of serious AEs, while sex showed no significant association (P = 0.162). ARIA-E and cerebral haemorrhage were more likely to be reported as severe. In contrast, headache, fatigue, chills and pyrexia were more likely to be reported as non-serious (**Supplementary Figure 2**).

A total of 314 patients experienced ARIA AEs. The clinical characteristics of these patients are outlined in **Table 1**. The univariate analysis demonstrated that drug number was significantly associated with the occurrence of serious ARIA AEs (P<0.0001), while neither sex (P = 0.670) nor age (P = 0.713) showed a statistically significant relationship. In the multivariate logistic regression analysis adjusting for all three variables, drug number remained a significant predictor of serious ARIA AEs (P = 0.046). Sex (P = 0.371) and age (P = 0.397) continued to exhibit no significant associations in the adjusted model.

### 3.4 Sensitivity analysis

When using donanemab and aducanumab as a comparator, SDR were also been identified, such as headache, ARIA-E, ARIA-H, infusion related reaction, confusional state, ARIA, fall, cerebral haemorrhage, gait disturbance, seizure, brain oedema, atrial fibrillation, disorientation, cerebral microhaemorrhage (**Supplementary Table 5**; **Supplementary Table 6**). Given that we speculate that the number of patients using aducanumab and donanemab is limited, AE reports should be interpreted cautiously.

### 3.5 Characteristics of the multicenter cohort population

A total of 148 patients were included in this study. Baseline characteristics of this population are shown in **Table 4**. The median age was 67 years, and 68.92% (102/148) were females. The median MMSE score was 21. Regarding the genetic profile, approximately half of the patients were carriers of ApoE ϵ4 allele, with 4.05% being homozygous and 45.95% heterozygous. Additionally, the median number of discharge diagnoses was eight and the median aCCI score was four. Patients concurrently used a median of five medications alongside lecanemab. The dosage of lecanemab varied among patients, ranging from 380 mg to 990 mg, with a median dosage of 600 mg.

**Table 4:** General characteristics of the multicenter cohort studied population.

Characteristics	Total(n = 148)
Gender	
Female	102(68.92)
Male	46(31.08)
Age, y	67.00(60.00, 74.00)
Hight, cm	160.00(156.25, 168.00)
Weight, kg	60.00(54.25, 68.75)
MMSE score	21(17, 23)
MoCA score	16(12, 19)
History of smoking	15(10.14)
History of drinking	10(6.76)
Family history of cognitive impairment	33(22.29)
History of allergies	14(9.46)
ApoE ϵ4 status	
Homozygote	6(4.05)
Heterozygote	68(45.95)
Noncarrier	65(43.92)
Dosage of lecanemab	600(550, 670)
Number of diagnoses at discharge	8(6, 12)
aCCI	4(3, 5)
Number of drugs used simultaneously	5(4, 7.75)

Categorical variables were expressed as n (percentage). Continuous variables did not follow a normal distribution and were expressed as M (P25, P75). MMSE, Mini-Mental State Examination; MoCA, Montreal Cognitive Assessment; ApoE, apolipoprotein E; aCCI, aged-adjusted Charlson comorbidity index.

### 3.6 Occurrence and influencing factors of AE

Among the study population, 43 (29.05%) patients experienced AEs. As shown in **Table 5**, the most common reported AE was infusion-related reactions, with symptoms primarily including pyrexia, chills, nausea, and vomiting. All infusion related reactions occurred during the first dose administration and manifested within 12 hours post-infusion, with an average onset time of 6.27 hours. These reactions resolved after an average of 11.43 hours (ranging from 1 to 60 hours) following symptomatic treatment or no intervention. In patients who did not receive preventative medication, the incidence of infusion-related reactions was 28.57% (4 of 14). In comparison, the rate of infusion-related reactions dropped to 24.63% (33 of 134) among those who received preventative medication.

**Table 5:** The occurrence of adverse events related to lecanemab.

Adverse events	Lecanemab(n = 148) no(%)
Adverse events related to lecanemab	43(29.05)
Serious adverse events*	5(3.38)
Adverse events leading to drug withdrawal	1(0.67)
Infusion related reaction	37(25.00)
Pyrexia	36(24.32)
Chills	4(2.70)
Nausea	2(1.35)
Vomiting	2(1.35)
Limb discomfort	1(0.67)
Dizziness	3(2.03)
ARIA-H	2(1.35)
Headache	1(0.67)
Atrial fibrillation	1(0.67)
Ventricular extrasystoles†	1(0.67)
Electrocardiogram QT prolonged†	1(0.67)
Chest discomfort†	1(0.67)
Hypersensitivity	1(0.67)
Tremor†	1(0.67)
Fall	1(0.67)

ARIA-H, amyloid-related imaging abnormality with cerebral microhaemorrhage, cerebral macrohaemorrhages, or superficial siderosis.

*Serious adverse events included 3 cases of infusion related reactions, 1 case of atrial fibrillation, and 1 case of ventricular extrasystoles and electrocardiogram QT prolonged, all of which prolonged the hospital stay.

†Adverse events that were not mentioned in clinical trials or drug instructions.

Dizziness was the second most common AE, reported by three patients. Two patients experienced ARIA-H; both cases were asymptomatic. One case with APOE ϵ4 heterozygote was identified on Magnetic Resonance Imaging (MRI) before the fifth dose and continued with treatment, while the other with APOE ϵ4 homozygote was identified before the fourteenth dose, leading to a temporary suspension of treatment. ARIA-H occurred in 2 of 32 patients (6.25%) who received at least 4 lecanemab infusions and underwent at least 1 monitoring MRI. Five patients experienced serious adverse events (SAEs), including three cases of infusion related reactions, one case of atrial fibrillation, and one case of ventricular extrasystoles and electrocardiogram QT prolonged with infusion related reaction. All SAEs resulted in prolonged hospital stays. A case report of the new SAE is presented in the **Supplementary Material**.

Univariate analysis was conducted to identify factors associated with the occurrence of AEs, with results presented in **Supplementary Table 7**. Among the factors examined, only the use of preventative medication was found to be significantly different between the groups (P = 0.0265), while no other factors reached statistical significance. 134 (90.54%) patients received preventative medication for AEs, and 36 (26.87%) experienced AEs. Among the 14 patients (9.46%) who did not receive preventative medication, 8 (57.14%) experienced AEs. Multivariate analysis (**Supplementary Table 8**) revealed that age was a risk factor for AE occurrence [OR (95% CI): 1.109 (1.011–1.215), P = 0.028]. Preventative medication using was found to significantly reduce the incidence of AEs [OR (95% CI): 0.098 (0.021–0.457), P = 0.003]. In addition, the use of concomitant psychotropic medications showed no statistically significant difference in the occurrence of AEs between the two groups (**Supplementary Table 9**).

### 3.7 Network pharmacological analysis

**Figure 2:**
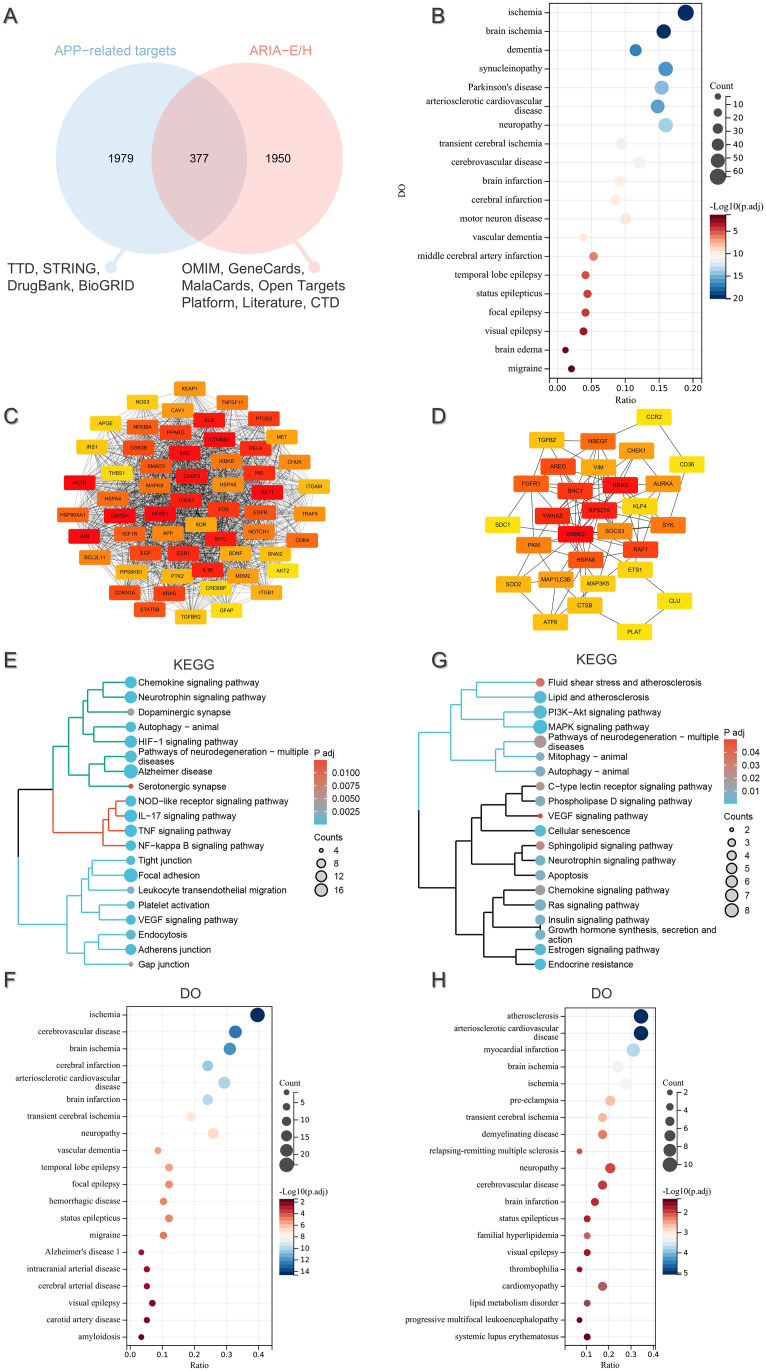
The lecanemab-ARIA-E/H potential target set (L-A set): drug-target gene interaction network analysis. **(A)** The Venn diagram illustrating the analysis approach of DTI network analysis. **(B)** The visualisation of the DO enrichment results of the L-A set. **(C, D)** The visualisation of the MCODE analysis results of the DTI network: subnetwork modules 1 and 2. The colors range from yellow to red, indicating a gradual increase in network degree values. **(E–H)** The visualisation of the KEGG and DO enrichment analysis results of subnetwork modules 1-2.

As shown in **Figure 2A**, the L-A set comprised 377 targets. The L set included 2,356 targets distributed across four drug-target databases: BioGRID (2,325 targets), DrugBank (38 targets), TTD (7 targets), and STRING (11 targets). The AE set contained 2,327 targets retrieved from five disease databases, with the following distribution: CTD (1,779 targets), GeneCards (1,175 targets), MalaCards (103 targets), OMIM (98 targets), and Open Targets Platform (282 targets).

The DTI network constructed from the L-A set is presented in **Supplementary Figure 3A**. DO enrichment analysis of the DTI network revealed significant associations with brain ischemia, dementia, neuropathy, transient cerebral ischemia, cerebral infarction, and status epilepticus (**Figure 2B**). KEGG pathway analysis demonstrated enrichment in Fluid shear stress and atherosclerosis, Alzheimer’s disease, and Pathways of neurodegeneration-multiple diseases. GO enrichment results demonstrated that the biological processes were primarily classified into two categories. The first category was related to glial cell development and the regulation of neuronal death, and the second category was associated with cellular responses to peptides and biomolecular metabolism. The cellular components were mainly divided into three groups. Category 1 was linked to the interaction between cells and the extracellular matrix. Category 2 was connected with nerve cells. And category 3 was associated with platelet alpha granules. The molecular functions were predominantly categorized into two categories. Category 1 involved kinase activity, and category 2 was related to the binding of biomolecules such as amyloid-beta, Tau protein, lipoprotein particles, etc. (**Supplementary Figures 3B–E**).

MCODE analysis of the DTI network revealed eight subnetwork modules. We selected two subnetwork modules with more than 10 nodes and a score above six for further analysis (**Figures 2C, D**). For subnetwork module 1 (score 44.35, 58 nodes, 1264 edges), KEGG enrichment analysis showed that its pathways were mainly divided into three categories. Category 1 was associated with pathways like Alzheimer’s disease, pathways of neurodegeneration-multiple diseases, and Neurotrophin signaling pathway. Category 2 was linked to pathways such as IL-17, TNF, and NF-κB. Category 3 was related to pathways including Focal adhesion, Adherens junction and VEGF. DO enrichment analysis indicated that it was mainly connected with diseases like cerebrovascular disease, status epilepticus, and AD (**Figures 2E, F**). For subnetwork module 2 (score 6.29, 29 nodes, 88 edges), KEGG enrichment analysis revealed that its pathways were primarily divided into two categories. One category was associated with pathways like MAPK, PI3K-AKT, and Autophagy-animal, while the other was linked to pathways such as Endocrine resistance, Cellular senescence, and Neurotrophin. DO enrichment analysis suggested that it was mainly related to diseases such as brain ischemia, status epilepticus, and thrombophilia (**Figures 2G, H**). GO enrichment results for each subnetwork are presented in **Supplementary Figure 3**, **Supplementary Figure 4**, and **Supplementary Figure 5**.

## 4 Discussion

While AEs of lecanemab have been reported in clinical trials (11, 30), real-world evidence regarding its safety profile is still needed, particularly in diverse ethnic populations and patients with complex comorbidities. In addition, it remains unclear the mechanisms underlying the AEs caused by lecanemab, especially ARIA. To our knowledge, this is the first study integrating both pharmacovigilance analysis of the FAERS database, multicenter observational cohort data and network pharmacology to systematically evaluate post-marketing AEs of lecanemab.

The common SDR included headache, ARIA-E, ARIA-H, chills, and infusion-related reactions, consistent with our previous FAERS study (13). Of 41 positive SDRs (≥4 cases), 12 were classified as important, ARIA-E and ARIA-H received the maximum priority score. In addition, 75% (9/12) of these prioritized AEs fell within nervous system disorders, predominantly comprising ARIA, reflecting their substantial pharmacovigilance significance. Notably, we identified advancing age and the number of medications as an independent predictor of serious AEs. This contrasts with our prior FAERS analysis, which found no age (p=0.076) or sex (p=0.448) associations, possibly due to underpowered subgroup stratification (13). These insights confirm sex does not affect serious AE risk while highlighting the imperative for targeted investigations into geriatric risk stratification and medication burden management strategies.

The timing of AEs is a key focus, as it helps clinicians identify critical periods for close monitoring. The WSP analysis revealed that ARIA-E, headache, and infusion-related reactions occurred early in treatment. Notably, status epilepticus showed a duration-dependent risk escalation, whereas ARIA-H, cerebral haemorrhage was no clear relationship with timing of treatment initiation. These findings consistent with the Clarity AD (Core+OLE) trial (11). In Clarity AD (Core+OLE) trial, ARIA-E predominantly occurred within the first 3–6 months. In contrast, the timing of isolated ARIA-H events was at a steady rate across the treatment course at the same rate as placebo. For intracerebral hemorrhage (ICH), including cerebral haemorrhage, there was likewise no clear relationship with timing of treatment initiation. However, the limited numbers for certain AEs (e.g., cerebral haemorrhage, n=28) may constrain the reliability of these temporal patterns.

We identified several AEs not reported in regulatory trials (or noted in < 5% of patients), such as tremors, seizures and ischaemic stroke. Tremor is a defining feature of Parkinson’s disease and it has also been identified in our multicenter cohort analysis. ARIA may present with a rare incidence of serious and life-threatening events, including seizure. A case report documented concurrent ischemic strokes and seizures in a patient presenting with ARIA-characteristic microhemorrhages and cerebral edema, suggests that ischemic strokes may also be a feature of ARIA (31). However, due to the small sample size, the SDR is not robust and needs to be further verified.

Lecanemab treatment was well tolerated with discontinuation in only 0.67% of patients in multicenter cohort analysis. The incidence rate of infusion-related reactions in our study similar to the Clarity AD (Core+OLE) trial (11) and Paczynski et al. (32). We also revealed an ARIA-H incidence rate of 6.25%, consistent with the 6.7% reported by Paczynski et al. and approaching the 9.1% observed in the Clarity AD (Core+OLE) trial. While no ARIA-E cases were found in our cohort, Paczynski et al. identified ARIA-E occurrence in 15% of patients. This discrepancy may stem from the markedly lower proportion of APOE ϵ4 homozygotes in our population (4.5%) compared to the 16% prevalence observed by Paczynski et al. It has been known that isolated ARIA-H demonstrates a correlation with microhemorrhage at baseline than with APOE ϵ4 genotype (P = 0.32), whereas ARIA-E was more common in ApoE ϵ4 carriers, with the highest frequency in homozygotes (32). Biologically, APOE ϵ4 carriers exhibit elevated parenchymal and cerebrovascular Aβ burden, which predisposes them to enhanced therapeutic Aβ clearance and increased vascular permeability (33). The underrepresentation of APOE ϵ4 homozygotes in our cohort likely reflects risk-adapted clinical decision-making, wherein heightened ARIA risk in this homozygote’s subgroup deters therapeutic initiation of lecanemab among patients, caregivers and clinicians.

The strong FAERS signals for ARIA-E/H versus the low occurrence in our cohort (no ARIA-E, two ARIA-H cases) reflect several factors. First, as shown in **Supplementary Table 4**, the number of ARIA reports for all other drugs in FAERS is very low, indicating that these events are extremely rare in the general population. This sparse data inflates RORs, meaning the strong signals represent reporting disproportionality rather than high real-world incidence. Second, ARIA-E is highly dependent on APOE ϵ4 homozygosity, which was present in only 4.5% of our Chinese cohort. Third, incomplete MRI follow-up (only 21.6% had adequate surveillance) may have underestimated asymptomatic ARIA.

While infusion-related reactions occurred among patients who did not receive a preventative medication more frequently than reported in the Clarity AD (Core+OLE) trial (11), their incidence decreased following protocol modifications incorporating antihistamine and acetaminophen pretreatment prior to initial infusions. Nonetheless, given the non-randomized, observational nature of this study, the apparent protective effect of preventative medication should be interpreted cautiously, as premedication decisions may reflect physician preference, hospital practice, or underlying patient risk. It is worth noting that real-world clinical practice demonstrates that clinicians often administering prophylactic premedication (e.g., oral antihistamines and acetaminophen) prior to the first infusion, despite guidelines recommending such interventions only after an initial reaction occurs (34–37). For example, the ADRD-TWG recommendations suggest that patients receive premedication with oral diphenhydramine 25–50 mg and oral acetaminophen 650–1,000mg 30 minutes prior to the next infusion following an infusion reaction. To some extent, this reflects that the concerns clinicians about infusion-related reaction, although it was almost mild.

The target genes in the DTI network demonstrated significant enrichment in DO entries such as Alzheimer’s disease type 1, dementia, and neuropathy, consistent with lecanemab’s pharmacological action on APP. Concurrently, these gene sets were also enriched in entries associated with brain edema, brain ischemia, cerebrovascular disease, hemorrhagic diseases, thrombophilia, and status epilepticus, suggesting their potential involvement in lecanemab-related AEs including ARIA-E, ARIA-H, ischemic stroke, and epilepsy.

The monoclonal antibodies bound to Aβ, along with large amounts of Aβ, move through the perivascular clearance pathway. As large amounts of Aβ enter the blood vessels, it triggers inflammation in the surrounding arteries, weakening the cohesion of the vessel walls. This increases the permeability of the vessel walls, leading to edema and microhemorrhages (38–41). Mechanistically, network pharmacology analysis indicates that ARIA-E pathogenesis may involve lecanemab-induced perturbations in signaling pathways including VEGF, Leukocyte transendothelial migration, Focal adhesion, Adherens junction, Tight junction, and Lipid and atherosclerosis. The VEGF, Leukocyte transendothelial migration, Focal adhesion, Adherens junction, and Tight junction signaling pathways have been reported to regulate cerebral microvascular blood flow, mediate endothelial responses to vasoactive mediators, and promote leukocyte migration or adhesion, thereby weakening the restrictive capacity of blood-brain barrier (BBB) against blood-derived component infiltration into the brain. Furthermore, the Lipid and atherosclerosis signaling pathway participates in modulating central nervous system lipid metabolism, thereby influencing BBB integrity (42–44). Collectively, these pathways may alter cerebrovascular permeability and BBB integrity, leading to vascular leakage and subsequent acute cerebral edema, which aligns with our findings from FAERS analysis and the Clarity AD (Core+OLE) trial. Similarly, ARIA-H-related microhemorrhages likely result from vascular permeability alterations mediated by these signaling pathways, along with erythrocyte extravasation.

Additionally, our FAERS analysis results revealed that lecanemab may potentially trigger AEs such as ischemic stroke, seizures, and status epilepticus. Current studies indicate that ischemic stroke is one of the clinical manifestations of ARIA (31). The pathogenesis of ischemic stroke may be associated with platelet activation and thrombosis mediated by signaling pathways including HIF-1, Platelet activation, Ras, and Phospholipase D (45–48). The mechanisms underlying epilepsy may involve neuronal hyperexcitability, abnormal electrical activity, dysregulated dopaminergic or 5-HT systems, and abnormal intercellular electrical coupling mediated by signaling pathways such as Dopaminergic synapse, Serotonergic synapse, Gap junction, Insulin, and Neurotrophin (49–53). Furthermore, we observed significant enrichment of signaling pathways including IL-17, TNF, NF-κB, NOD-like receptor, MAPK, and Chemokine in both subnetwork modules, suggesting that AD-related neuroinflammation and inflammatory responses may contribute to the development of ARIA-E, ARIA-H, ischemic stroke, and epilepsy (54–57). However, the potential roles of these signaling pathways as mechanisms underlying lecanemab-related AEs during AD treatment require further molecular biological investigations for validation.

There are several limitations of our study. First, the FAERS database is a worldwide spontaneous reporting system. It has some inherent selection bias, such as voluntary (underreporting), the inability to establish causality, reporting bias, data quality issues. Nevertheless, it remains the largest accessible post-marketing pharmacovigilance resource, and we ensure the reliability of the results as much as possible through strict deduplication method and standardized SDR scoring framework. Second, the sample size of the multicenter cohort was small, and the observational study had selection bias and unmeasured confounders. The incidence of AEs such as ARIA may have been underestimated due to unavailability of information on visits to all medical facilities for each patient and the short follow-up period. Third, our cohort was derived from five tertiary hospitals. Patients treated in such settings may have better access to specialist care, which could limit the generalizability of our findings to primary care populations. However, because lecanemab prescription and monitoring require amyloid PET confirmation, infusion facilities, and specialized ARIA monitoring, it is currently primarily prescribed and monitored in tertiary hospitals in China. Therefore, our findings remain relevant to the current real-world practice in China. Future population-based studies are needed to confirm external validity. Fourth, current understanding of disease-related protein interactions primarily originates from public databases, while drug-gene interaction studies lack experimental validation *in vitro* and *in vivo*.

In conclusion, this study leverages multimodal approaches-spanning pharmacovigilance analytics, real-world cohort studies, and molecular enrichment analyses-to provide a comprehensive assessment of lecanemab’s post-marketing safety profile. These findings provide crucial real-world evidence to define lecanemab’s safety risks. We advocate for such integrated analyses of real-world evidence to become a standard component of post-marketing safety evaluation, necessitating further validation through large-scale prospective studies.

## Data Availability

The original contributions presented in the study are included in the article/**Supplementary Material**. Further inquiries can be directed to the corresponding authors.
