# Supplementary material for: Post-marketing safety of lecanemab: a real-world study based on FAERS database, multicenter cohort and network pharmacology
Source: Front Psychiatry. 2026 Jun 22;17:1822543. doi: 10.3389/fpsyt.2026.1822543 (PMC13333610; doi:10.3389/fpsyt.2026.1822543)
Supplement: Supplementary file 1 [file SupplementaryFile1.docx]

***Post-marketing safety of lecanemab: a real-world study based on FAERS database, multicenter cohort and network pharmacology***

**Supplemental Material**

[Supplemental Table 1. A rating scale assessing clinical priority of disproportionality signals. 6](#_Toc230079822)

[Supplemental Table 2. Clinical priority assessing results of SDR 7](#_Toc230079823)

[Supplemental Table 3. SDRs of lecanemab for adverse events with more than four counts. 11](#_Toc230079824)

[Supplemental Table 4. Two‑by‑two contingency tables for ARIA‑E and ARIA‑H in the FAERS database 15](#_Toc230079825)

[Supplemental Table 5. SDRs of donanemab for adverse events with more than four counts. 16](#_Toc230079826)

[Supplemental Table 6. SDRs of aducanumab for adverse events with more than four counts. 17](#_Toc230079827)

[Supplemental Table 7. Univariate analysis of related factors associated with adverse events occurrence of lecanemab. 19](#_Toc230079828)

[Supplemental Table 8. Multivariable analysis of related factors associated with adverse events occurrence of lecanemab. 20](#_Toc230079829)

[Supplemental Table 9. Association between concomitant psychotropic medications and adverse events in patients receiving lecanemab 21](#_Toc230079830)

[Supplemental Figure 1. Time to onset of adverse events (A) difference between male and female and (B) difference among different ages of lecanemab. 22](#_Toc230079831)

[Supplemental Figure 2. Volcano map of difference between serious and non-serious adverse events of lecanemab. 23](#_Toc230079832)

[Supplemental Figure 3. The lecanemab-ARIA-E/H potential target network and enrichment analyses. 24](#_Toc230079833)

[Supplemental Figure 4. Enrichment analyses of subnetwork module 1. 25](#_Toc230079834)

[Supplemental Figure 5. Enrichment analyses of subnetwork module 2. 26](#_Toc230079835)

[A case report of the new serious adverse event related to lecanemab. 27](#_Toc230079836)

**Methods**

**Data Source and Data Processing**

The FDA Adverse Event Reporting System (FAERS) is a spontaneous database that contains adverse events (AEs) reports, medication error reports and product quality complaints resulting in AEs that were submitted to the FDA.^1^ Healthcare professionals, consumers, and manufacturers submit reports to FAERS. The FAERS database supports post-marketing safety surveillance for drug and therapeutic biologic products, currently containing 21 million spontaneously reported AE cases. The AEs are coded using terms in the Medical Dictionary for Regulatory Activities (MedDRA) terminology.^2,3^ These coded terms are arranged in a hierarchy of 5 categories that include broad (system organ class [SOC]) and specific categories (e.g., preferred term [PT]).

In the FAERS database, the reported AEs and the indications of drugs are coded according to the Medical Dictionary for Regulatory Activities (MedDRA 27.0). The reported medication exposure data will be standardised using the World Health Organization Drug Dictionary (March 2024). We extracted the data from FAERS on February 1, 2025. In addition, we excluded duplicate reports according to FDA guidelines to ensure accuracy. Our study was exempt from institutional review board approval or informed patient consent. Patients are further deduplicated if they meet: (1) identical demographics/timing (gender, age, weight, event/medication dates); (2) adverse reactions occurring before treatment; (3) ≥50 reported reactions per patient; or (4) non-drug-related adverse event terms.

**Statistical analysis**

We used two disproportionality approaches to increase consistency and robustness of findings. We estimated the reporting odds ratio (ROR) and the Bayesian information component (IC) for all AEs with at least 4 reports. The ROR method is easier to understand and analyze than the Bayesian one and is the most widely used.^4^ Bayesian approaches may be more effective with increased confounding or larger effect sizes.^4^

A semiquantitative scoring system incorporating five key parameters was implemented to prioritize signal of disproportionate reporting (SDR): number of target events, the lower limit of the 95% confidence interval of the ROR (ROR_025_), mortality proportion, important medical events (IMEs) or designated medical events (DMEs), relevant evidence evaluation.^5^ Based on composite scores (0-10), AEs were stratified into three clinical priority tiers: weak (0-4), moderate (5-7), and strong (8-10).

Time-to-onset (TTO) intervals were calculated as the duration between lecanemab initiation (START_DT) and adverse event manifestation (EVENT_DT). Medians, quartiles, and the Weibull shape parameter (WSP) test were used to assess the TTO. The WSP test was applied to analyze the pattern of AE incidence over time. The Weibull distribution is characterized by 2 parameters: scale (a) and shape (b). The shape parameter (β) determines the hazard trend over time: if β < 1 with a 95% confidence interval (CI) entirely < 1, the hazard decreases (early failure type); if β approximates 1 with a 95% CI contains the value 1, the hazard remains constant (random failure type); and if β > 1 with a 95% CI entirely > 1, the hazard increases over time (wearout failure type).^6,7^

Univariate logistic regression was performed to assess associations between age, sex, number of medications, and serious AEs. Proportions were analyzed using chi-square (χ2) tests, while the Mann-Whitney U test (M-W-U) was applied for age comparisons. A multivariate model adjusted for all three variables identified independent predictors. Data manipulation and statistical analyses were performed using SAS software (version 9.4)

**Reference**

1. US Food and Drug Administration. Questions and answers on FDA’s Adverse Event Reporting System. https://www.fda.gov/drugs/surveillance/questions-and-answers-fdas-adverse-event-reporting-system-faers. Accessed September 25, 2024.

2.MedDRA. Introductory Guide: MedDRA version 27.1. https://admin.new.meddra.org/sites/default/files/guidance/file/intguide_27_1_English.pdf. Published September 2024. Accessed September 25, 2024.

3.ICH. Harmonisation for better health: MedDRA work products. http://www.ich.org/products/meddra.html. Accessed September 25, 2024.

4.Dijkstra L, Garling M, Foraita R, Pigeot I. Adverse drug reaction or innocent bystander? A systematic comparison of statistical discovery methods for spontaneous reporting systems. Pharmacoepidemiol Drug Saf. 2020;29(4):396-403. doi:10.1002/pds.4970.

5.Gatti M, Antonazzo IC, Diemberger I , et al. Adverse events with sacubitril/valsartan in the real world: emerging signals to target preventive strategies from the FDA adverse event reporting system. Eur J Prev Cardiol, 2021;28(9): p. 983-989. doi: 10.1177/2047487320915663

6. Kinoshita S, Hosomi K, Yokoyama S, et al. Time‐to‐onset analysis of amiodarone‐associated thyroid dysfunction. J Clin Pharm Ther. 2020;45(1):65–71. doi: 10.1111/jcpt.13024

7. Mazhar F, Battini V, Gringeri M, et al. The impact of anti-TNFα agents on weight-related changes: new insights from a real-world pharmacovigilance study using the FDA adverse event reporting system (FAERS) database. Expert Opin Biol Ther. 2021;21(9):1281–1290. doi: 10.1080/14712598.2021.1948529

# **Supplemental Table 1. A rating scale assessing clinical priority of disproportionality signals.**

| Assessment items | 0 point | 1 point | 2 points |
| --- | --- | --- | --- |
| Number of target events | <10 | 10-50 | >50 |
| ROR_025_ | 1-2 | 2-5 | >5 |
| Mortality proportion | <25% | 25-50% | >50% |
| IMEs or DMEs | - | IME | DME |
| Relevant evidence evaluation | - | + | ++ |

Mortality Proportion: The percentage of cases where death was reported as an outcome among the total cases reported for a specific adverse event (AE).

IMEs (Important Medical Events) and DMEs (Designated Medical Events): These classifications are developed and periodically updated by the European Medicines Agency (EMA, 2020).

Evidence Ratings: ++: AEs are primarily sourced from FDA Prescribing Information, the Summary of Product Characteristics of lecanemab provided by the MHRA, Phase 2/3 randomized controlled trials (RCTs), or systematic reviews, with established biological plausibility. +: AEs are primarily sourced from other clinical trials, observational studies, or case reports/series with potential biological plausibility. -: AEs identified exclusively through disproportionality analyses.

Abbreviations: AEs: Adverse Events, DMEs: Designated Medical Events, IMEs: Important Medical Events, MHRA: Medicines and Healthcare Products Regulatory Agency, RCTs: Randomized Controlled Trials, ROR_025_: The lower limit of the 95% confidence interval of the Reporting Odds Ratio.

# **Supplemental Table 2. Clinical priority assessing results of SDR**

| PTs | n | ROR_025_ | Death(n) | IME/DME | Relevant evidence evaluation | Priority level (score) |
| --- | --- | --- | --- | --- | --- | --- |
| Amyloid related imaging abnormality-oedema/effusion | 190 | 16701.18 | 9 | IMEs | ++ | moderate(7) |
| Amyloid related imaging abnormality-microhaemorrhages and haemosiderin deposits | 164 | 16987.36 | 8 | IMEs | ++ | moderate(7) |
| Headache | 234 | 6.75 | 0 | NA | ++ | moderate(6) |
| Chills | 138 | 17.93 | 1 | NA | ++ | moderate(6) |
| Infusion related reaction | 120 | 29.6 | 2 | NA | ++ | moderate(6) |
| Cerebral haemorrhage | 28 | 9.29 | 6 | IMEs | ++ | moderate(6) |
| Brain oedema | 15 | 12.42 | 1 | IMEs | ++ | moderate(6) |
| Status epilepticus | 5 | 4.95 | 3 | IMEs | ++ | moderate(6) |
| Pyrexia | 82 | 3.55 | 1 | NA | ++ | moderate(5) |
| Amyloid related imaging abnormalities | 48 | 15844.35 | 0 | NA | ++ | moderate(5) |
| Seizure | 22 | 2.14 | 3 | IMEs | ++ | moderate(5) |
| Cerebral microhaemorrhage | 9 | 273.3 | 0 | IMEs | ++ | moderate(5) |
| Confusional state | 80 | 7.26 | 4 | NA | - | weak(4) |
| Nausea | 76 | 1.38 | 3 | NA | ++ | weak(4) |
| Dizziness | 66 | 1.83 | 1 | NA | ++ | weak(4) |
| Influenza like illness | 27 | 3.79 | 0 | NA | ++ | weak(4) |
| Syncope | 18 | 1.92 | 0 | IMEs | ++ | weak(4) |
| Atrial fibrillation | 12 | 1.57 | 1 | IMEs | ++ | weak(4) |
| Magnetic resonance imaging abnormal | 8 | 14.25 | 0 | NA | ++ | weak(4) |
| Superficial siderosis of central nervous system | 7 | 1018.63 | 1 | NA | ++ | weak(4) |
| Fatigue | 105 | 2.04 | 0 | NA | - | weak(3) |
| Vomiting | 48 | 1.39 | 0 | NA | ++ | weak(3) |
| Fall | 30 | 1.07 | 4 | NA | ++ | weak(3) |
| Gait disturbance | 22 | 1.36 | 0 | NA | ++ | weak(3) |
| Feeling cold | 20 | 8.08 | 0 | NA | - | moderate(3) |
| Cerebral infarction | 10 | 4.45 | 0 | IMEs | - | weak(3) |
| Subdural haematoma | 9 | 5.36 | 1 | IMEs | - | weak(3) |
| Ischaemic stroke | 7 | 4.62 | 0 | IMEs | + | weak(3) |
| Tremor | 47 | 3.95 | 0 | NA | - | weak(2) |
| Disorientation | 11 | 2.58 | 1 | NA | - | weak(2) |
| Aphasia | 10 | 2.97 | 1 | NA | - | weak(2) |
| Brain fog | 7 | 7.36 | 0 | NA | - | weak(2) |
| Body temperature increased | 5 | 1.73 | 0 | NA | ++ | weak(2) |
| Somnolence | 27 | 1.68 | 1 | NA | - | weak(1) |
| Balance disorder | 12 | 1.43 | 0 | NA | - | weak(1) |
| Head discomfort | 8 | 4.08 | 0 | NA | - | weak(1) |
| Infusion site extravasation | 4 | 4.07 | 0 | NA | - | weak(1) |
| Dizziness postural | 4 | 2.9 | 0 | NA | - | weak(1) |
| Incontinence | 4 | 2.57 | 0 | NA | - | weak(1) |
| Lethargy | 8 | 1.18 | 0 | NA | - | weak(0) |
| Heart rate decreased | 7 | 1.59 | 1 | NA | - | weak(0) |

Note: SOC: system organ class, SDR: Signals of disproportionate reporting, PTs: preferred terms, DMEs: designated medical events, lMEs: important medical events, ROR_025_: The lower limit of the 95% confidence interval of the Reporting Odds Ratio. n: number of cases, NA: not designated medical events or important medical events.

# **Supplemental Table 3. SDRs of lecanemab for adverse events with more than four counts.**

| PT | No. | ROR | Lower  95%CI | Upper 95%CI | IC | Lower 95%CI | Upper 95%CI | death | serious AE | Time To Event  (median(Q1,Q3)) |
| --- | --- | --- | --- | --- | --- | --- | --- | --- | --- | --- |
| Headache | 234 | 7.72 | 6.75 | 8.82 | 2.84 | 2.80 | 2.60 | 0 | 28 | 0.00(0.00,1.00) |
| ARIA-E | 190 | 20828.19 | 16701.18 | 25975.02 | 13.02 | 7.54 | 7.28 | 9 | 111 | 49.00(40.00,76.00) |
| ARIA-H | 164 | 21601.29 | 16987.36 | 27468.42 | 13.05 | 7.34 | 7.05 | 8 | 83 | 48.00(35.00,79.00) |
| Chills | 138 | 21.28 | 17.93 | 25.26 | 4.34 | 4.15 | 3.90 | 1 | 21 | 0.00(0.00,0.00) |
| Infusion related reaction | 120 | 35.54 | 29.60 | 42.69 | 5.09 | 4.74 | 4.47 | 2 | 53 | 0.00(0.00,1.00) |
| Fatigue | 105 | 2.48 | 2.04 | 3.01 | 1.28 | 1.26 | 0.97 | 0 | 14 | 0.00(0.00,1.00) |
| Pyrexia | 82 | 4.43 | 3.55 | 5.51 | 2.11 | 2.06 | 1.73 | 1 | 13 | 0.00(0.00,0.00) |
| Confusional state | 80 | 9.07 | 7.26 | 11.33 | 3.14 | 3.01 | 2.68 | 4 | 23 | 1.00(0.00,32.00) |
| Nausea | 76 | 1.74 | 1.38 | 2.18 | 0.78 | 0.77 | 0.43 | 3 | 15 | 0.00(0.00,0.00) |
| Dizziness | 66 | 2.33 | 1.83 | 2.98 | 1.20 | 1.17 | 0.82 | 1 | 15 | 0.00(0.00,4.00) |
| Vomiting | 48 | 1.85 | 1.39 | 2.46 | 0.87 | 0.85 | 0.43 | 0 | 12 | 0.00(0.00,0.00) |
| ARIA | 48 | 25290.04 | 15844.35 | 40366.84 | 13.16 | 5.61 | 5.08 | 0 | 17 | 42.50(32.00,55.00) |
| Tremor* | 47 | 5.27 | 3.95 | 7.03 | 2.38 | 2.26 | 1.84 | 0 | 7 | 0.00(0.00,0.00) |
| Fall | 30 | 1.54 | 1.07 | 2.20 | 0.61 | 0.59 | 0.07 | 4 | 24 | 10.00(0.00,33.00) |
| Cerebral haemorrhage | 28 | 13.49 | 9.29 | 19.58 | 3.74 | 3.23 | 2.69 | 6 | 29 | 54.00(27.00,177.00) |
| Influenza like illness | 27 | 5.53 | 3.79 | 8.08 | 2.46 | 2.24 | 1.69 | 0 | 2 | 0.00(0.00,0.50) |
| Somnolence | 27 | 2.46 | 1.68 | 3.59 | 1.29 | 1.22 | 0.67 | 1 | 5 | 0.00(0.00,1.00) |
| Gait disturbance | 22 | 2.06 | 1.36 | 3.14 | 1.04 | 0.97 | 0.37 | 0 | 4 | 0.50(0.00,6.50) |
| Seizure* | 22 | 3.26 | 2.14 | 4.96 | 1.70 | 1.56 | 0.96 | 3 | 31 | 35.00(29.00,57.00) |
| Feeling cold | 20 | 12.54 | 8.08 | 19.47 | 3.64 | 3.01 | 2.38 | 0 | 3 | 0.00(0.00,0.00) |
| Syncope | 18 | 3.05 | 1.92 | 4.84 | 1.60 | 1.45 | 0.79 | 0 | 8 | 0.00(0.00,71.00) |
| Brain oedema | 15 | 20.63 | 12.42 | 34.28 | 4.36 | 3.21 | 2.48 | 1 | 11 | 45.50(30.00,62.00) |
| Atrial fibrillation | 12 | 2.78 | 1.57 | 4.89 | 1.47 | 1.28 | 0.48 | 1 | 14 | 69.50(31.00,87.00) |
| Balance disorder | 12 | 2.52 | 1.43 | 4.45 | 1.33 | 1.17 | 0.37 | 0 | 4 | 0.00(0.00,3.00) |
| Disorientation | 11 | 4.67 | 2.58 | 8.45 | 2.22 | 1.84 | 1.00 | 1 | 4 | 0.00(0.00,0.00) |
| Aphasia | 10 | 5.52 | 2.97 | 10.28 | 2.46 | 1.97 | 1.09 | 1 | 2 | 44.50(32.00,57.00) |
| Cerebral infarction | 10 | 8.28 | 4.45 | 15.41 | 3.04 | 2.31 | 1.44 | 0 | 10 | 68.00(49.00,84.00) |
| Subdural haematoma | 9 | 10.31 | 5.36 | 19.84 | 3.36 | 2.41 | 1.50 | 1 | 6 | 62.00(28.00,182.00) |
| Cerebral microhaemorrhage | 9 | 532.08 | 273.30 | 1035.90 | 9.00 | 3.30 | 2.36 | 0 | 5 | 25.00(0.00,211.00) |
| Head discomfort | 8 | 8.18 | 4.08 | 16.37 | 3.03 | 2.18 | 1.22 | 0 | 0 | 0.00(0.00,0.00) |
| Lethargy | 8 | 2.36 | 1.18 | 4.73 | 1.24 | 1.03 | 0.07 | 0 | 0 | 0.00(0.00,0.00) |
| Magnetic resonance imaging abnormal | 8 | 28.55 | 14.25 | 57.19 | 4.83 | 2.81 | 1.85 | 0 | 1 | 146.00(49.00,262.00) |
| Heart rate decreased | 7 | 3.33 | 1.59 | 7.00 | 1.73 | 1.37 | 0.34 | 1 | 3 | 0.00(0.00,14.00) |
| Ischaemic stroke* | 7 | 9.70 | 4.62 | 20.37 | 3.27 | 2.21 | 1.19 | 0 | 8 | 44.00(36.00,47.00) |
| Superficial siderosis of central nervous system | 7 | 2260.71 | 1018.63 | 5017.34 | 10.93 | 2.99 | 1.90 | 1 | 5 | 40.00(31.00,42.00) |
| Brain fog | 7 | 15.46 | 7.36 | 32.47 | 3.95 | 2.46 | 1.44 | 0 | 4 | 0.00(0.00,0.00) |
| Body temperature increased | 5 | 4.15 | 1.73 | 9.98 | 2.05 | 1.44 | 0.26 | 0 | 0 | 0.00(0.00,0.00) |
| Status epilepticus | 5 | 11.90 | 4.95 | 28.61 | 3.57 | 2.08 | 0.90 | 3 | 10 | 49.50(42.00,57.00) |
| Dizziness postural | 4 | 7.74 | 2.90 | 20.65 | 2.95 | 1.72 | 0.43 | 0 | 0 | 0.00(0.00,0.00) |
| Incontinence | 4 | 6.85 | 2.57 | 18.27 | 2.77 | 1.66 | 0.37 | 0 | 0 | 0.00(0.00,34.00) |
| Infusion site extravasation | 4 | 10.87 | 4.07 | 28.99 | 3.44 | 1.87 | 0.58 | 0 | 0 | 14.00(14.00,14.00) |

* Unexcepted SDR

Abbreviations: SDR, Signals of disproportionate reporting; PT, Preferred Terms; CI, confidence interval; ROR, reporting odds ratio; IC, information component; ARIA, Amyloid related imaging abnormalities; ARIA-E, amyloid-related imaging abnormality with oedema/effusion; ARIA-H, amyloid-related imaging abnormality with cerebral microhaemorrhage, cerebral macrohaemorrhages, or superficial siderosis.

# **Supplemental Table 4. Two‑by‑two contingency tables for ARIA‑E and ARIA‑H in the FAERS database**

| Adverse event | Drug exposure | AE reports | Other reports |
| --- | --- | --- | --- |
| ARIA‑E | Lecanemab | 190 | 2,574 |
|  | Other drugs | 142 | 40,067,740 |
| ARIA‑H | Lecanemab | 164 | 2,600 |
|  | Other drugs | 117 | 40,067,765 |

Notes: AE reports = number of reports for the specific adverse event (ARIA‑E or ARIA‑H); Other reports = number of adverse event reports for all other events in the same drug exposure group. Data source: FDA Adverse Event Reporting System (FAERS), 2023–2024.*

# **Supplemental Table 5. SDRs of donanemab for adverse events with more than four counts.**

| PT | No. | ROR | Lower  95%CI | Upper 95%CI | IC | Lower 95%CI | Upper 95%CI |
| --- | --- | --- | --- | --- | --- | --- | --- |
| Headache | 11 | 8.65 | 4.65 | 16.10 | 2.99 | 1.45 | 3.20 |
| Amyloid related imaging abnormality-microhaemorrhages and haemosiderin deposits | 10 | 13818.76 | 7149.62 | 26708.85 | 13.57 | 2.53 | 4.38 |
| Amyloid related imaging abnormality-oedema/effusion | 8 | 32.72 | 15.96 | 67.09 | 4.93 | 1.83 | 3.83 |
| Flushing | 8 | 9076.95 | 4391.12 | 18763.12 | 13.01 | 2.16 | 4.18 |
| Infusion related reaction | 7 | 49.73 | 23.16 | 106.77 | 5.55 | 1.74 | 3.85 |
| Amyloid related imaging abnormalities | 5 | 25195.21 | 9984.74 | 63576.85 | 14.46 | 1.33 | 3.84 |
| Cerebral microhaemorrhage | 4 | 5651.06 | 2068.87 | 15435.70 | 12.39 | 0.99 | 3.65 |

Abbreviations: SDR, Signals of disproportionate reporting; PT, Preferred Terms; CI, confidence interval; ROR, reporting odds ratio; IC, information component

# **Supplemental Table 6. SDRs of aducanumab for adverse events with more than four counts.**

| PT | No. | ROR | Lower  95%CI | Upper 95%CI | IC | Lower 95%CI | Upper 95%CI |
| --- | --- | --- | --- | --- | --- | --- | --- |
| Amyloid related imaging abnormality-oedema/effusion | 134 | 31532.22 | 25073.18 | 39655.16 | 13.99 | 6.76 | 7.37 |
| Amyloid related imaging abnormality-microhaemorrhages and haemosiderin deposits | 97 | 23549.14 | 18257.92 | 30373.77 | 13.76 | 6.25 | 6.96 |
| Headache | 39 | 3.41 | 2.47 | 4.69 | 1.73 | 1.18 | 2.11 |
| Confusional state | 33 | 10.44 | 7.38 | 14.77 | 3.34 | 2.49 | 3.50 |
| Cerebral haemorrhage | 25 | 34.00 | 22.86 | 50.58 | 5.05 | 3.32 | 4.46 |
| Fall | 22 | 3.17 | 2.08 | 4.83 | 1.64 | 0.90 | 2.12 |
| Superficial siderosis of central nervous system | 22 | 30230.85 | 17376.64 | 52593.86 | 14.06 | 3.80 | 5.25 |
| Seizure | 18 | 7.49 | 4.70 | 11.94 | 2.88 | 1.80 | 3.13 |
| Amyloid related imaging abnormalities | 17 | 11817.27 | 6864.78 | 20342.66 | 13.14 | 3.41 | 4.93 |
| Brain oedema | 13 | 50.10 | 28.98 | 86.62 | 5.63 | 2.69 | 4.25 |
| Atrial fibrillation | 11 | 7.12 | 3.93 | 12.91 | 2.82 | 1.39 | 3.07 |
| Gait disturbance | 11 | 2.88 | 1.59 | 5.22 | 1.52 | 0.47 | 2.14 |
| Cognitive disorder | 11 | 12.05 | 6.65 | 21.84 | 3.58 | 1.80 | 3.48 |
| Memory impairment | 9 | 3.14 | 1.63 | 6.06 | 1.64 | 0.45 | 2.28 |
| Urinary tract infection | 9 | 2.66 | 1.38 | 5.13 | 1.40 | 0.27 | 2.10 |
| COVID-19 | 9 | 3.11 | 1.62 | 6.00 | 1.63 | 0.44 | 2.27 |
| Subarachnoid haemorrhage | 8 | 37.53 | 18.71 | 75.29 | 5.22 | 1.92 | 3.86 |
| Head injury | 7 | 10.62 | 5.05 | 22.34 | 3.40 | 1.24 | 3.29 |
| Cerebral microhaemorrhage | 7 | 1145.89 | 539.24 | 2435.03 | 10.11 | 1.95 | 4.03 |
| Transient ischaemic attack | 5 | 8.01 | 3.33 | 19.28 | 2.99 | 0.70 | 3.06 |
| Vertigo | 5 | 3.94 | 1.63 | 9.48 | 1.97 | 0.22 | 2.58 |
| Mental status changes | 5 | 8.45 | 3.51 | 20.36 | 3.07 | 0.73 | 3.09 |
| Disorientation | 4 | 4.73 | 1.77 | 12.62 | 2.24 | 0.14 | 2.73 |

Abbreviations: SDR, Signals of disproportionate reporting; PT, Preferred Terms; CI, confidence interval; ROR, reporting odds ratio; IC, information component

# **Supplemental Table 7. Univariate analysis of related factors associated with adverse events occurrence of lecanemab.**

| Variable | Non-AE occurrence group (N=105) | AE occurrence group (N=43) | *P* |
| --- | --- | --- | --- |
| Gender |  |  | 0.887 |
| Male | 33(31.43) | 13(30.23) |  |
| Female | 72(68.57) | 30(69.77) |  |
| Age | 67.00(60.00, 73.00) | 67.00(58.00, 77.00) | 0.527 |
| ApoE ε4 status |  |  | 0.524 |
| Homozygous | 3(2.86) | 3(6.98) |  |
| Heterozygous | 49(46.67) | 19(44.19) |  |
| Noncarrier | 46(43.81) | 19(44.19) |  |
| Dosage of lecanemab | 580(530, 600) | 600(560, 710) | 0.055 |
| Number of diagnoses at discharge | 9(6, 13) | 8(5, 11) | 0.199 |
| aCCI | 4(3, 5) | 4(2, 5) | 0.891 |
| Number of drugs used simultaneously | 5(4, 7) | 4(3, 8) | 0.271 |
| Preventative medications using | 99(94.29) | 35(81.40) | 0.027 |

Categorical variables were expressed as n (percentage). Continuous variables did not follow a normal distribution and were expressed as M (P25, P75).

ApoE: apolipoprotein E; aCCI: aged-adjusted Charlson comorbidity index.

# **Supplemental Table 8. Multivariable analysis of related factors associated with adverse events occurrence of lecanemab.**

| Variable | β | SE | OR(95%CI) | *P* |
| --- | --- | --- | --- | --- |
| Gender(female) | -0.1840 | 0.2979 | 0.692(0.215-2.225) | 0.537 |
| Age | 0.1030 | 0.0470 | 1.109(1.011-1.215) | 0.028 |
| ApoE ε4 status |  |  |  | 0.294 |
| Homozygous | 0.8447 | 0.5907 | 3.069(0.520-18.125) | 0.153 |
| Heterozygous | -0.5680 | 0.3703 | 0.747(0.321-1.739) | 0.125 |
| Dosage of lecanemab | 0.00424 | 0.00237 | 1.004(1.000-1.009) | 0.074 |
| Number of diagnoses at discharge | -0.0216 | 0.0434 | 0.979(0.899-1.066) | 0.620 |
| aCCI | -0.6666 | 0.3579 | 0.513(0.255-1.036) | 0.063 |
| Number of drugs used simultaneously | 0.0414 | 0.0675 | 1.042(0.913-1.119) | 0.540 |
| Preventative medications using | -1.1606 | 0.3926 | 0.098(0.021-0.457) | 0.003 |

ApoE: apolipoprotein E; aCCI: aged-adjusted Charlson comorbidity index.

# **Supplemental Table 9. Association between concomitant psychotropic medications and adverse events in patients receiving lecanemab**

| Concomitant medications | Non-AE occurrence group (N=105) | AE occurrence group (N=43) | *P* |
| --- | --- | --- | --- |
| Antidepressants |  |  | 0.181 |
| Yes | 25(23.81) | 6(13.95) |  |
| No | 80(76.19) | 37(86.05) |  |
| Antipsychotics |  |  | 0.487 |
| Yes | 3(2.86) | 3(6.98) |  |
| No | 102(97.14) | 40(93.02) |  |
| Benzodiazepines |  |  | 0.423 |
| Yes | 12(11.43) | 7(16.28) |  |
| No | 93(88.57) | 36(83.72) |  |

Categorical variables were expressed as n (percentage).


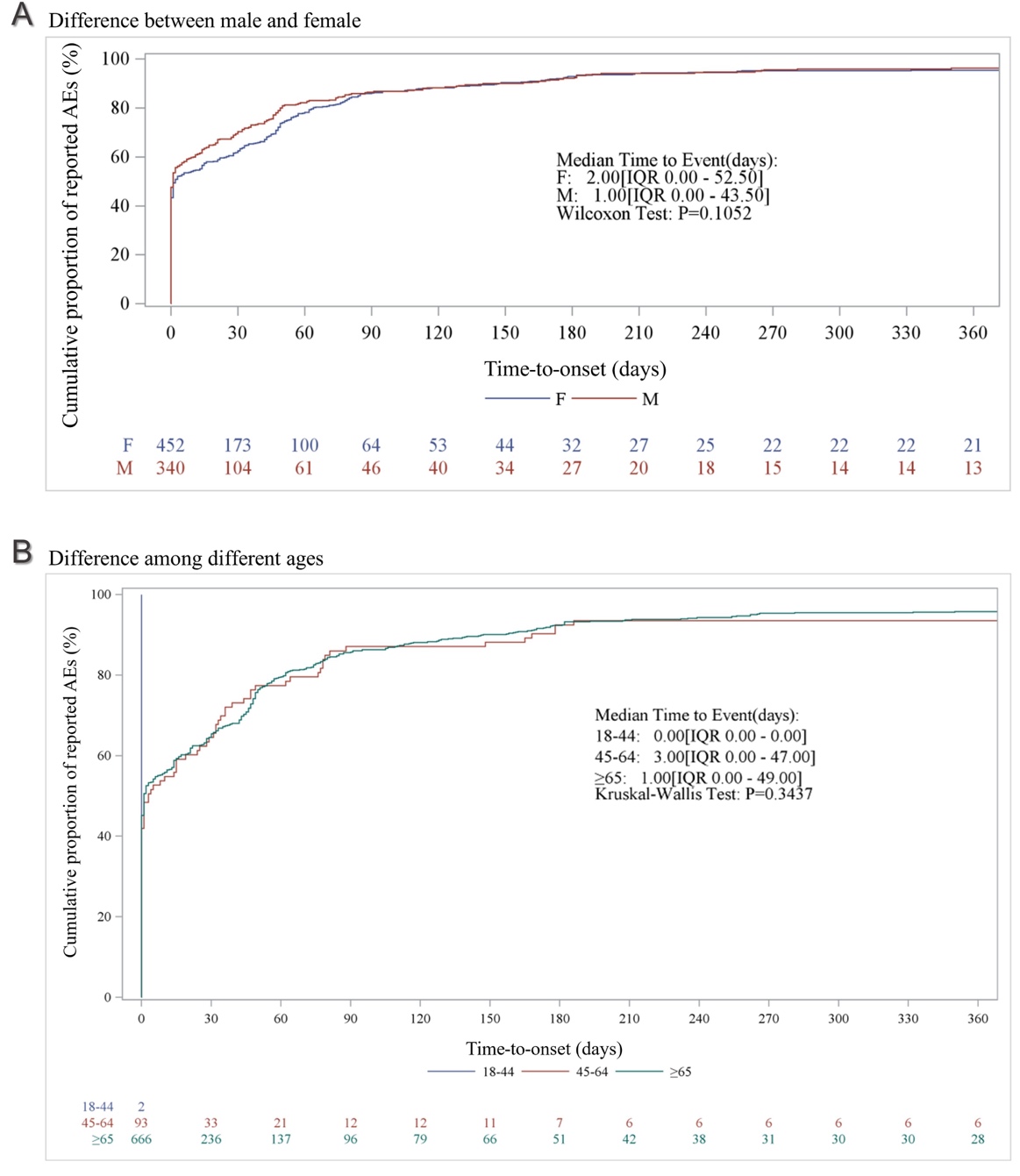


# **Supplemental Figure 1. Time to onset of adverse events (A) difference between male and female and (B) difference among different ages of lecanemab.**

**
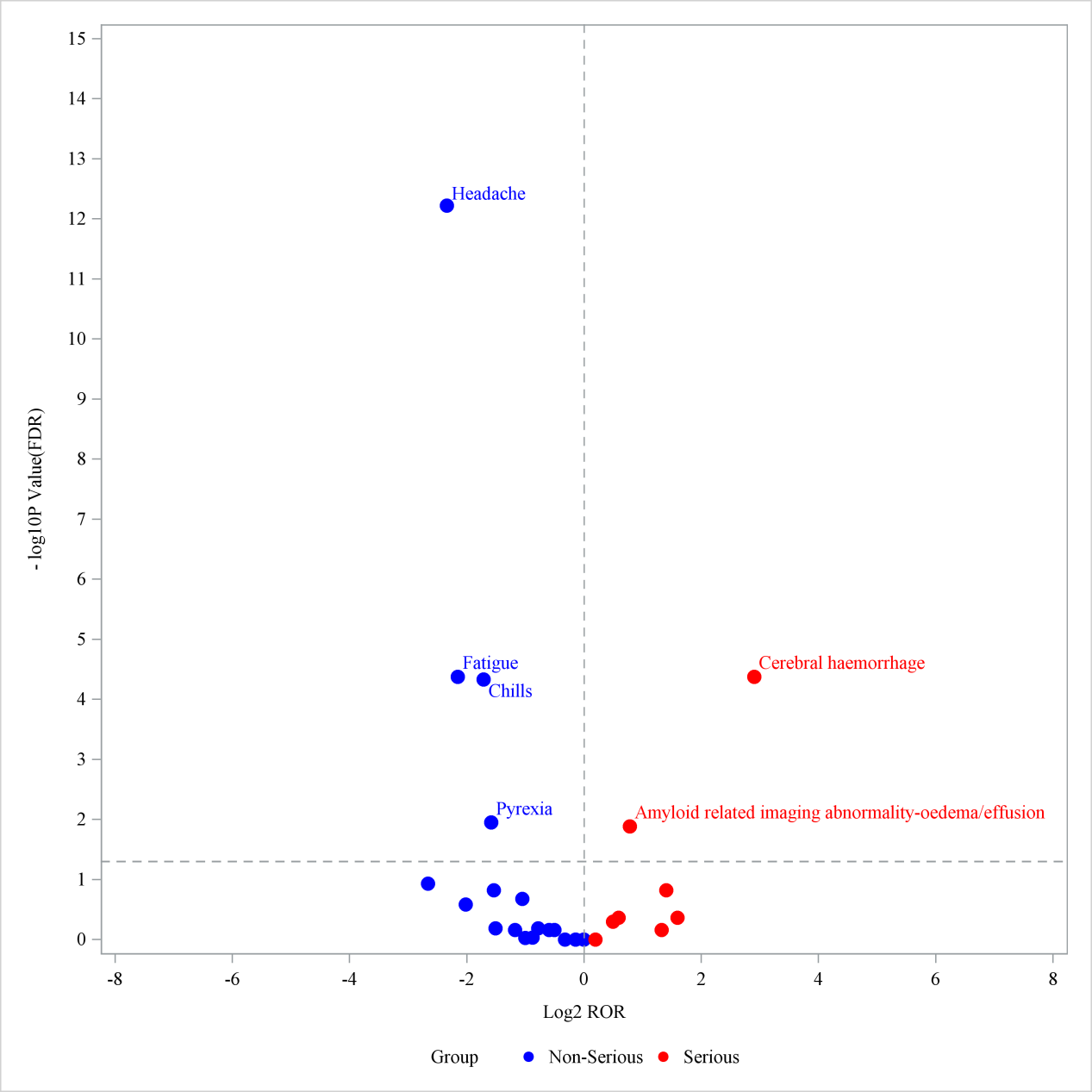
**

# **Supplemental Figure 2. Volcano map of difference between serious and non-serious adverse events of lecanemab.**


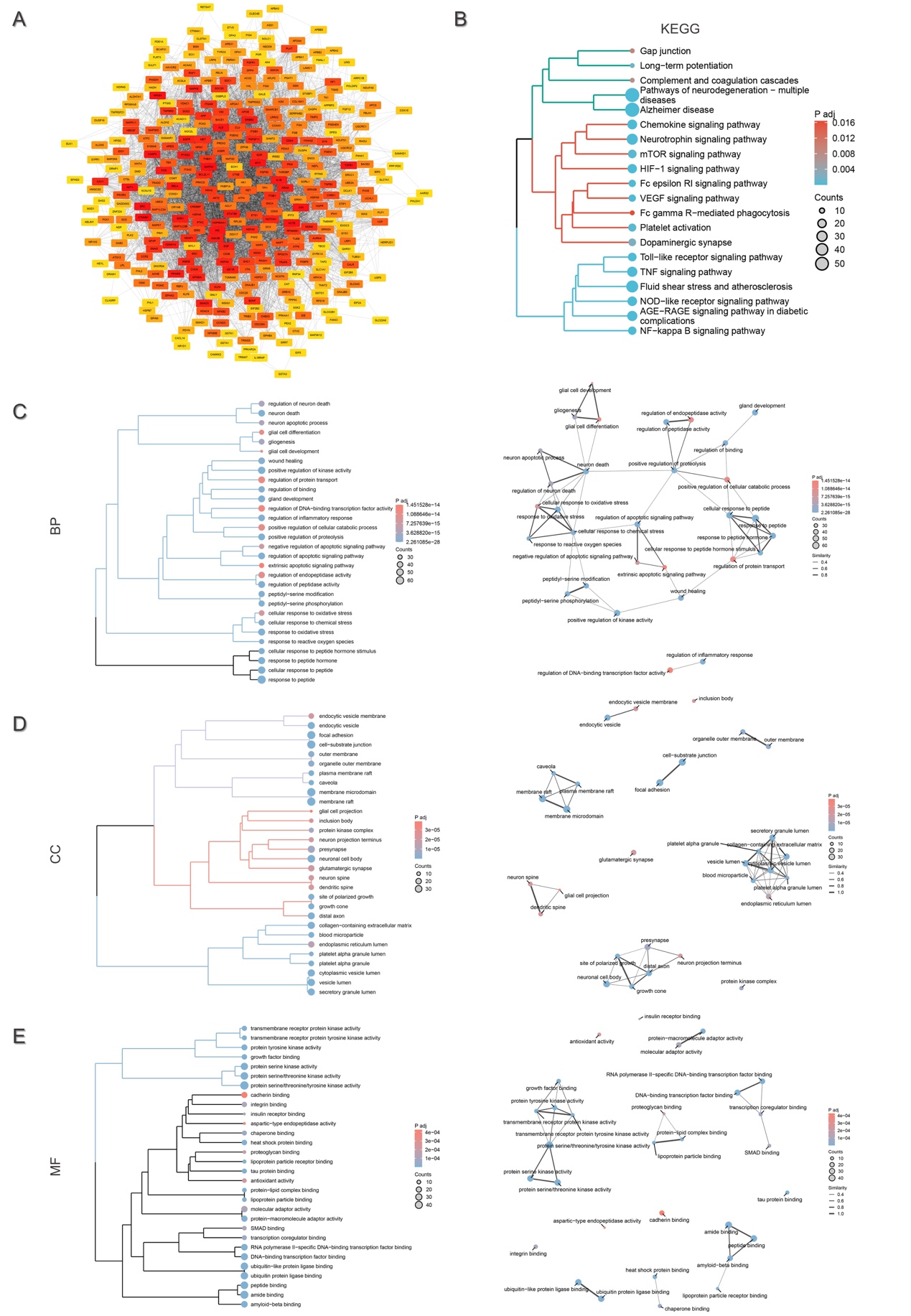


# **Supplemental Figure 3. The lecanemab-ARIA-E/H potential target network and enrichment analyses.**


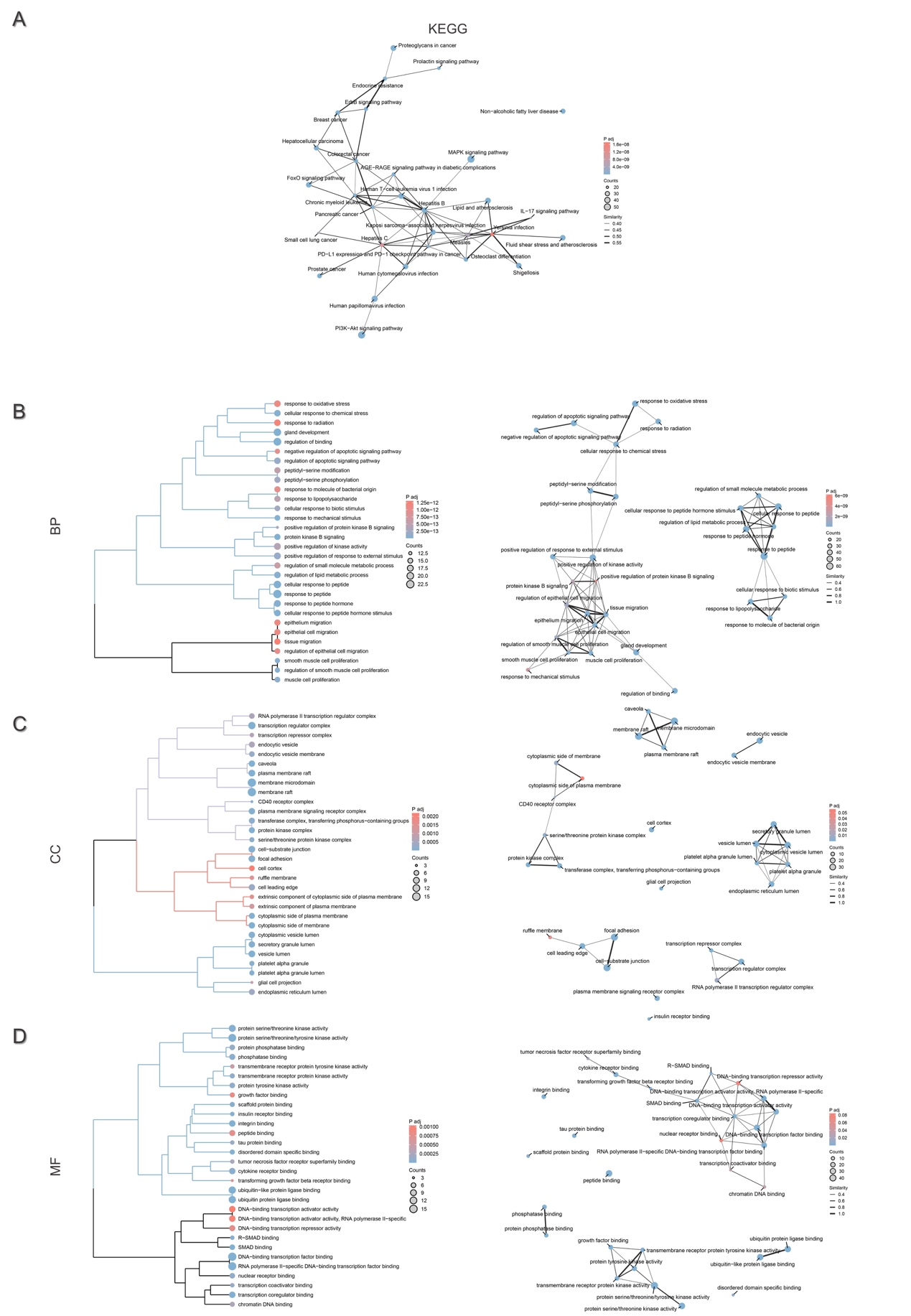


# **Supplemental Figure 4. Enrichment analyses of subnetwork module 1.**


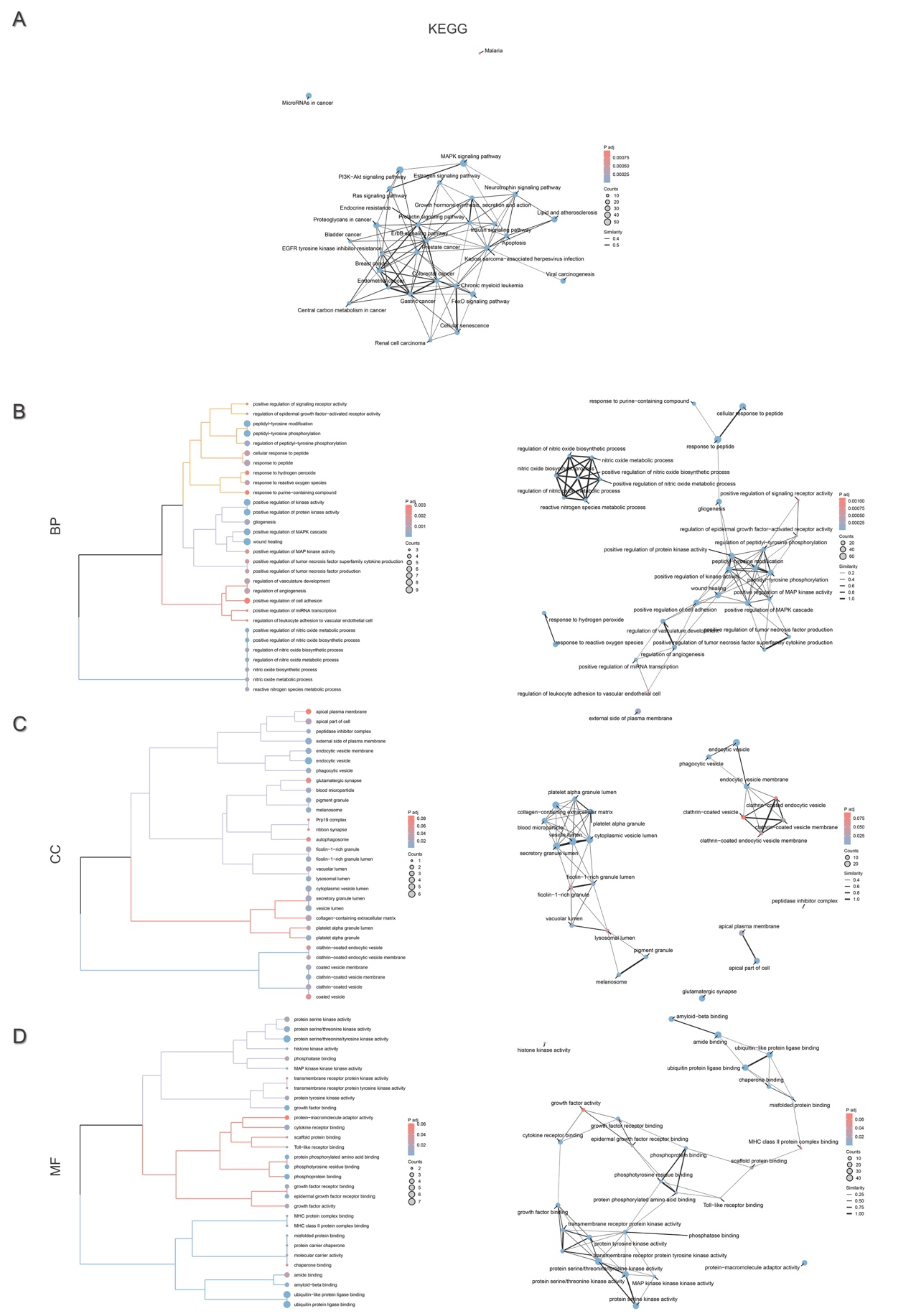


# **Supplemental Figure 5. Enrichment analyses of subnetwork module 2.**

# **A case report of the new serious adverse event related to lecanemab.**

The patient is an 81-year-old female, with a height of 147 cm and a weight of 63 kg. She visited the clinic in March 2024. A 18F-AV45 PET/CT scan revealed increased radiotracer uptake in the bilateral frontal, parietal, and temporal cortices, with more pronounced findings on the left side. This was considered indicative of a positive Aβ (amyloid-beta) imaging result, leading to a diagnosis of Alzheimer's disease.

The patient has experienced a decline in hearing in her left ear. Over a decade ago, she had a "fracture of the thoracic vertebrae", for which no specific treatment was administered. She has had panniculitis in both legs for 10 years. In 2011, she underwent a "pulmonary nodule resection". In 2016, she had a hip replacement surgery. In November 2023, she suffered a thalamic infarction. Her current medications include atorvastatin calcium tablets 20 mg at night (qn) and clopidogrel 75 mg once daily (qd). She has no history of food or drug allergies. She does not smoke and does not consume alcohol. She is married, but her spouse has passed away. She has two daughters who are in good health.

On December 30, 2024, at 13:43, the patient received an intravenous infusion of 600 mg lecanemab. Thirty minutes before the infusion, diphenhydramine was administered to prevent allergic reactions. The infusion was completed at 14:49. The patient reported numbness of the tongue, chest pain, and radiation to the back. Her vital signs were as follows: temperature 37.3°C, pulse 83 beats per minute, and respiratory rate 23 breaths per minute. An urgent electrocardiogram (ECG) showed sinus rhythm, ventricular premature beats, nonspecific T-wave changes, and prolonged QT interval. One tablet of nitroglycerin was administered sublingually, and oxygen was provided via nasal cannula at 2 L/min. The patient's cardiac enzyme levels and BNP (B-type natriuretic peptide) results returned without significant abnormalities. She vomited a large amount of gastric contents and was given an intramuscular injection of metoclopramide and an intravenous infusion of pantoprazole. At 18:00, the patient's temperature rose to 38.2°C, and by 21:58, it had decreased to 37.9°C. A bedside examination revealed no palpitations, chest tightness, or chest discomfort, and vomiting had ceased.

The patient's pyrexia, vomiting, and arrhythmia, were considered likely related to the use of lecanemab, with a causality rating of "probable." The patient's temperature and vital signs were closely monitored. On December 31, 2024, at 10:00, the patient's temperature dropped to 37°C. On January 3, 2025, at 10:45, the patient was alert and articulate, and a neurological examination by a neurologist revealed no significant positive findings.
